# Differential impact of manic versus depressive episode recurrence on longitudinal gray matter volume changes in bipolar disorder

**DOI:** 10.1038/s41386-025-02197-x

**Published:** 2025-08-15

**Authors:** Florian Thomas-Odenthal, Lea Teutenberg, Frederike Stein, Nina Alexander, Linda M. Bonnekoh, Katharina Brosch, Kira Flinkenflügel, Janik Goltermann, Dominik Grotegerd, Tim Hahn, Andreas Jansen, Elisabeth J. Leehr, Susanne Meinert, Julia-Katharina Pfarr, Harald Renz, Kai Ringwald, Navid Schürmeyer, Thomas Stief, Benjamin Straube, Katharina Thiel, Paula Usemann, Axel Krug, Igor Nenadić, Udo Dannlowski, Tilo Kircher

**Affiliations:** 1 Philipps-Universität Marburg, Faculty of Medicine, Department for Psychiatry and Psychotherapy, Marburg, Germany; 2 Philipps-Universität Marburg, Center for Mind, Brain, and Behavior (CMBB), Marburg, Germany; 3 Institute for Translational Psychiatry, University of Münster, Münster, Germany; 4 Department of Child and Adolescent Psychiatry, University Hospital Münster, Münster, Germany; 5 Institute of Behavioral Science, Feinstein Institutes for Medical Research, Manhasset, NY USA; 6 Core-Facility BrainImaging, Faculty of Medicine, University of Marburg, Marburg, Germany; 7 Institute for Translational Neuroscience, University of Münster, Münster, Germany; 8 Department of Neurology and Neurosurgery, McGill University, Montreal, Canada; 9 Institute of Laboratory Medicine, Philipps University of Marburg, Marburg, Germany; 10 Department of Psychiatry and Psychotherapy, University Hospital of Bonn, Bonn, Germany; 11 Bielefeld University, Medical School and University Medical Center OWL, Protestant Hospital of the Bethel Foundation, Department of Psychiatry, Bielefeld, Germany

**Keywords:** Bipolar disorder, Neuroscience

## Abstract

Bipolar disorder (BD) is a severe mental disorder, characterized by episodes of mania and depression. The longitudinal neurobiological impact of BD episodes on brain structure remains largely unknown. In 124 age-sex-matched participants (62 BD patients; 62 healthy controls; HCs), aged 20-62 years, we investigated the longitudinal relationship between BD episodes and whole-brain gray matter volume (GMV) changes (3 Tesla MRI) during a two-year interval, using voxel-based morphometry in SPM12/CAT12. We compared GMV trajectories between BD patients with at least one depressive or manic episode during the two-year interval, BD patients without an episode, and HCs. We explored associations between GMV changes and clinical variables, like the number and duration of depressive or manic episodes both during the two-year interval and before baseline assessment. BD patients showed GMV increases in the right exterior cerebellum with an increasing number of depressive episodes during the two-year interval. BD patients without recurrence showed GMV reductions in this area, relative to BD patients with recurrence and HCs. Notably, BD patients without recurrence exhibited greater GMV reductions during the two-year interval, the longer they had spent in a manic episode before baseline. Our findings underscore the dynamic nature of brain changes in BD. GMV increases in BD patients with recurrence may be due to acute neuroinflammatory mechanisms including glial cell proliferation, whereas GMV reductions in BD patients without recurrence may result from abnormal synaptic refinement or pruning, as a consequence of past neuroinflammation during BD episodes.

## Introduction

Bipolar disorder (BD) is a complex mental disorder characterized by episodes of mania and depression, affecting ~1% of the global population [1]. Such episodes disrupt social and occupational functioning [2] and may alter brain structure [3].

Cross-sectional magnetic resonance imaging (MRI) studies show that BD patients have decreased gray matter volumes (GMVs) in the insula, thalamus, anterior cingulate cortex, fusiform gyrus, superior temporal gyrus, and inferior, middle, and superior frontal gyri, and increased GMVs in the putamen, precuneus, and posterior cingulate cortex, compared to healthy controls (HCs), according to recent meta-analyses [4–7]. Longitudinal MRI research extends these findings by demonstrating progressive GMV reductions over time in the frontal and temporal lobes and anterior cingulate cortex, compared to HCs [3, 8]. Longitudinal studies are crucial to determine whether GMV changes are a precursor to or a consequence of BD. However, longitudinal evidence from BD patients is scarce, inconsistent, and limited by small sample sizes, with different brain regions being affected, ranging from GMV decreases to increases [9], and in some cases no significant changes at all compared to HCs [10].

This inconsistency in brain structural findings may be due to several factors such as aging, genetics, pharmacological treatment, and the number and timing of mood episodes relative to scanning [3, 9]. Within the neuroprogression framework [11], particularly, mood episodes exert cumulative effects on brain structure through mechanisms like low-grade inflammatory processes [9], as indicated by markers such as C-reactive protein (CRP) [12, 13]. Low-grade peripheral inflammation may lead to neuroinflammation, excitotoxicity, and oxidative stress [14], particularly in areas critical for emotion regulation [14–16], which may sensitize the system to future stressors, triggering more frequent BD episodes [17], and leading to more GMV changes over time [14, 15]. During remission, these brain changes stabilize, leading to fewer GMV changes over time, similar to the age-related decline observed in HCs [9, 17]. These findings suggest a dynamic interplay between episodes, disease progression, and recovery in influencing GMV trajectories in BD.

Recent longitudinal studies have begun to explore the impact of recurrent BD episodes on brain structural changes over time. First-episode BD patients with at least one depressive or manic episode during the scanning interval showed GMV reductions in frontal, temporal, and parietal regions, compared to those without an episode and HCs [17]. BD patients with manic episodes during the interval showed gray matter decreases in frontal regions compared to those without an episode [18] and HCs [19]. In contrast, BD patients with an increasing number of depressive episodes showed gray matter increases in the cingulate gyrus, thalamus, and lenticular nuclei [20], alongside decreases in the temporal lobe [21, 22]. Notably, BD patients with an increasing number of manic episodes during the interval showed gray matter decreases in the cerebellum [21], while gray matter increases in the cerebellum were observed with an increasing number of depressive episodes [20]. These findings suggest a dynamic interplay between the episode polarity and GMV changes, even within the same region.

However, these previous studies often did not compare brain structural changes in BD patients with recurrence to those without recurrence or HCs, nor did they explore the impact of episode type (manic vs. depressive), or considered patients’ baseline episode history on brain structural changes. Investigating these factors is crucial to determine, whether the observed brain changes during a longer interval (years) reflect adaptive versus maladaptive neurobiological responses to recurrent BD episodes. By comparing GMV changes in BD patients with recurrence to those without recurrence and HCs, we could determine whether the observed brain changes resulted from BD episodes.

Therefore, for the first time, we elucidate GMV changes in groups of BD patients with and without a depressive or manic episode and HCs during a two-year follow-up interval within the neuroprogression framework. We hypothesized that BD patients with an episode during the two-year interval would show GMV decreases associated with manic episodes or GMV increases associated with depressive episodes, consistent with the existing literature. We also hypothesized that BD patients without an episode would not show such GMV changes during the two-year interval, similar to HCs.

Furthermore, to elucidate the effects of previous mood episodes – i.e., those that occurred before our baseline assessment – on brain changes during the two-year interval, we explored the relationships between GMV changes during the two-year interval and the number and duration of depressive or manic episodes prior to baseline. We also explored potential predictive associations between baseline CRP levels and GMV changes during the two-year interval, to better understand the neuroimmunology behind the observed GMV changes. Lastly, we explored the influence of potential confounders on GMV changes, including the number and duration of hospitalizations during the two-year interval, changes in symptom severity, global psycho-social functioning, familial genetic risk, comorbid psychiatric diagnoses, and medication use.

## Materials and methods

### Participants

124 age-sex-matched participants (*n* = 62 HC; *n* = 62 BD) were included in this analysis from the ongoing Marburg–Münster Affective Disorder Cohort Study (MACS) [23], which is part of the FOR2107 consortium investigating the neurobiology of major psychiatric disorders. All available data relevant to our research question were included in this analysis. Some of this data was previously analyzed in cross-sectional studies investigating GMV alterations in BD patients relative to HCs [24–27]. All participants underwent T1-weighted MRI scans and clinical assessments at both baseline (T1) and follow-up (T2) time points, approximately two years apart (mean = 2.18 years, SD = 0.26, range: 1.96–3.28 years). Assessments were conducted at the University of Marburg and the University of Münster in Germany. Inclusion criteria required that participants were between 18 and 65 years of age at baseline (T1) time point. Exclusion criteria were a history of neurological or general medical conditions, substance dependence, and verbal IQ ≤ 80. HCs were further excluded if they met the criteria for current or past mental disorders as per Structured Clinical Interview for DSM-IV-TR (SCID-I) [28], or if they had a lifetime intake of psychotropic medication (for details, see ref. [23]). To minimize potential overlaps in brain structural variance between BD patients and HCs, HCs were further excluded with a positive family history of BD and those with subsyndromal depressive or manic symptoms during the two-year follow-up interval (T2-T1) (Supplementary Materials and Methods S1). To ensure a balanced comparison between groups, HCs were matched 1:1 to BD patients by age and sex using the MatchIt package in R (version 4.3.1) [29], to avoid any confounding effects from these variables [30, 31]. This study was approved by the ethics committees of the medical faculties at the University of Marburg (AZ: 07/14) and the University of Münster (AZ: 2014-422-b-S) in accordance with the Declaration of Helsinki. Participants provided written informed consent and received financial compensation after receiving a full description of the study.

#### Assessment of clinical variables

Clinical variables were assessed through semi-structured interviews at both baseline (T1) and follow-up (T2) time points, including the course of illness (number and duration of depressive or manic episodes, number and duration of hospitalizations), current remission status (partially or fully remitted, according to SCID-I criteria), psychopathology (17-item Hamilton Depression Rating Scale [HAM-D], Young Mania Rating Scale [YMRS]) [32, 33], global psycho-social functioning (Global Assessment of Functioning; GAF) [34], familial risk (first-degree relative had been treated for BD, or treated for major depressive disorder [MDD], schizophrenia [SCZ], or schizoaffective disorder [SZA], considered together), body mass index (BMI), and current medication use, amongst others. Descriptive statistics of the study participants are presented in Table 1.

**Table 1:** Descriptive statistics of BD recurrence groups at baseline (T1) and follow-up (T2) time point.

	HC (*n* = 62)	BD non-recurrence (*n* = 23)	BD recurrence^a^ (*n* = 39)	*P*	BD whole sample (*n* = 62)	*P*^*b*^
Baseline (T1)						
Age	39.44 (12.24)	40.96 (11.72)	38.62 (12.13)	0.703	39.48 (11.94)	0.944
Sex, *n*	F = 31, M = 31	F = 12, M = 11	F = 19, M = 20	0.966	F = 31, M = 31	1.00
TIV	1527.19 (141.53)	1585.41 (134.86)	1573.93 (143.57)	0.299	1578.19 (139.39)	0.133
Education, years	14.45 (2.86)	14.65 (2.87)	14.67 (3.00)	0.959	14.66 (2.93)	0.785
BMI	24.71 (3.79)	28.43 (4.06)	27.17 (5.15)	0.002	27.62 (4.79)	0.001
First-degree relative with BD, *n* (%)	0 (0%)	4 (18%)	1 (3%)	<0.001	5 (8%)	0.022
First-degree relative with MDD, SCZ, or SZA, *n* (%)	13 (21%)	11 (48%)	17 (44%)	0.016	28 (45%)	0.004
BD subtype, *n*	–	I = 15, II = 8	I = 18, II = 21	0.149	I = 33, II = 29	–
Comorbid anxiety disorder, *n* (%)	–	5 (22%)	10 (26%)	0.731	15 (24%)	–
Comorbid eating disorder, *n* (%)	–	1 (4%)	2 (5%)	0.891	3 (5%)	–
Comorbid alcohol abuse, *n* (%)	–	3 (13%)	4 (10%)	0.740	7 (11%)	–
Comorbid cannabis abuse, *n* (%)	–	1 (4%)	3 (8%)	0.608	4 (6%)	–
Smokers, *n* (%)	8 (13%)	4 (19%)	8 (21%)	0.538	12 (20%)	0.273
HAM-D	1.48 (1.98)	5.09 (4.90)	6.28 (6.86)	<0.001	5.84 (6.19)	<0.001
YMRS	0.56 (1.13)	2.26 (2.88)	4.62 (5.57)	<0.001	3.74 (4.86)	<0.001
GAF	90.68 (7.39)	69.26 (13.10)	62.15 (12.68)	<0.001	64.79 (13.19)	<0.001
hsCRP, mg/l^c^	1.31 (1.58)	3.77 (5.37)	3.83 (7.65)	0.231	3.81 (6.84)	0.102
Antipsychotics, *n* (%)	–	11 (48%)	16 (41%)	0.605	27 (44%)	–
Antidepressants, *n* (%)	–	13 (57%)	15 (38%)	0.171	28 (45%)	–
Anticonvulsants, *n* (%)	–	7 (30%)	12 (31%)	0.978	19 (31%)	–
Lithium, *n* (%)	–	8 (35%)	10 (26%)	0.447	18 (29%)	–
Medication load index	–	2.78 (1.86)	2.31 (1.79)	0.300	2.48 (1.82)	–
Sackeim score	–	2.91 (2.76)	1.79 (1.66)	0.179	2.20 (2.18)	–
CPZ score	–	156.25 (310.17)	97.89 (172.70)	0.749	118.67 (230.20)	–
Remission status	–	a = 5, r = 18	a = 18, r = 21	0.092	a = 21, r = 41	–
Number of depressive episodes before T1	–	5.22 (2.28)	9.17 (8.76)	0.313	7.60 (7.18)	–
Duration of depressive episodes before T1 [months]	–	29.58 (24.36)	59.78 (75.94)	0.507	48.53 (63.29)	–
Number of manic episodes before T1	–	3.55 (2.30)	7.08 (8.76)	0.602	5.78 (7.28)	–
Duration of manic episodes before T1 [months]	–	8.60 (6.56)	22.86 (45.79)	0.323	17.58 (37.00)	–
Two-year follow-up (T2)						
Interscan interval, days	788.10 (101.62)	794.74 (84.91)	812.49 (94.12)	0.047	805.90 (90.52)	0.027
TIV	1527.36 (140.43)	1573.89 (134.56)*	1570.73 (144.24)*	0.453	1571.90 (139.62)*	0.210
BMI	25.43 (4.25)*	27.82 (5.00)	27.44 (4.83)	0.037	27.57 (4.85)	0.011
HAM-D	0.95 (1.56)	2.26 (2.67)*	5.95 (5.01)	<0.001	4.58 (4.63)	<0.001
YMRS	0.17 (0.53)*	1.13 (1.96)	4.03 (5.04)	<0.001	2.95 (4.38)	<0.001
GAF	89.59 (8.95)	78.83 (10.84)*	65.56 (13.60)	<0.001	70.48 (14.12)*	<0.001
Antipsychotics, *n* (%)	–	9 (39%)	14 (36%)	0.801	23 (37%)	–
Antidepressants, *n* (%)	–	12 (52%)	15 (38%)	0.297	27 (44%)	–
Anticonvulsants, *n* (%)	–	6 (26%)	11 (28%)	0.858	17 (27%)	–
Lithium, *n* (%)	–	5 (22%)	10 (26%)	0.731	15 (24%)	–
Medication load index	–	2.04 (1.66)	2.34 (1.95)	0.694	2.23 (1.84)	–
Sackeim score	–	2.05 (2.33)	1.41 (1.60)	0.483	1.68 (1.95)	–
CPZ score	–	140.76 (291.07)	98.39 (168.32)	0.831	114.36 (221.28)	–
Remission status	–	a = 0, r = 23*	a = 15, r = 24	<0.001	a = 15, r = 47	–
Number of depressive episodes between T1 and T2	–	0 (0)	1.62 (0.94)	<0.001	1.02 (1.08)	–
Duration of depressive episodes between T1 and T2 [months]	–	0.70 (1.89)	6.44 (5.45)	<0.001	4.31 (5.26)	–
Number of manic episodes between T1 and T2	–	0 (0)	0.64 (0.78)	<0.001	0.40 (0.69)	–
Duration of manic episodes between T1 and T2 [months]	–	0 (0)	1.35 (2.06)	<0.001	0.85 (1.75)	–
Number of hospitalizations between T1 and T2	–	0.13 (0.46)	0.51 (0.88)	0.023	0.37 (0.77)	–
Duration of hospitalizations between T1 and T2	–	0.52 (1.65)	1.06 (1.88)	0.046	0.86 (1.80)	–

All values are given as mean (SD) unless otherwise specified.

*P*-values stem from the non-parametric Kruskal–Wallis test for between-group comparisons or the Wilcoxon signed-rank test for within-group comparisons.

*a* acute, *r* partially or fully remitted (according to SCID-I/DSM-IV-TR), *BD* bipolar disorder, *BMI* body mass index, *CPZ* chlorpromazine equivalents, *GAF* Global Assessment of Functioning, *F* female, *M* male, *HAM-D* Hamilton Depression Rating Scale, *HC* healthy control, *hsCRP* high-sensitivity C-reactive protein, *I* BD subtype 1, *II* BD subtype 2, *MDD* major depressive disorder, *n* number of participants, *SCZ* schizophrenia, *SZA* schizoaffective disorder, *T1* baseline time point, *T2* follow-up time point, *TIV* total intracranial volume, *YMRS* Young Mania Rating Scale.

*Significant within-group differences between baseline and follow-up at *p* < 0.05; **Significant within-group differences between baseline and follow-up at *p* < 0.001.

^a^38 BD patients with recurrence (97%) had at least one depressive episode, and 18 patients with recurrence (46%) had at least one manic episode during the two-year interval.

^b^*P*-values stem from comparisons between BD and HC groups.

^c^hsCRP data was only available for 82 (66%) participants: HCs: *n* = 43 (69%); BD non-recurrence: *n* = 14 (61%); BD recurrence: *n* = 25 (64%).

### MRI acquisition and pre-processing

#### MRI acquisition

MRI data were acquired at both baseline (T1) and follow-up (T2) time points using a 3 Tesla MRI scanner (Siemens, Erlangen, Germany) with standardized pulse sequence parameters and extensive quality assurance protocols [35]. Details on acquisition parameters are provided in Supplementary Materials and Methods S2.

#### MRI preprocessing

T1-weighted scans were preprocessed using the longitudinal pipeline of the CAT12 toolbox (v1742; Structural Brain Mapping Group, Jena, Germany) in SPM12 (Institute of Neurology, London, UK) running under MATLAB (vR2017a, The MathWorks, Natick, Massachusetts, USA). Default preprocessing steps included realignment, bias correction, tissue classification, and spatial normalization using the Geodesic Shooting template. Images were segmented into gray matter, white matter, and cerebrospinal fluid, and smoothed with an 8 mm FWHM Gaussian kernel. Total intracranial volume (TIV) was calculated, and data were normalized to the Montreal Neurological Institute (MNI) space. Individual quality control included visual inspection and outlier identification using the check homogeneity function in CAT12.

#### Harmonization of imaging data

Imaging data from two sites (Marburg and Münster) and two body coil changes in Marburg were harmonized using the ComBat tool (v1.0.1) in MATLAB R2017a to correct for site- and scanner-related variations while preserving biological variability in the data (Supplementary Materials and Methods S3).

### Statistical analyses

#### Whole-brain analyses for longitudinal data

To investigate the effects of recurrent episodes in BD patients and GMV changes over time, and to compare these GMV changes with BD patients without episodes and HCs, we performed a 3 × 2 repeated measures Analysis of Covariance (ANCOVA) using the flexible factorial design in SPM12/CAT12. Subject, scanning time point (baseline [T1] and follow-up [T2]), and group (BD recurrence, BD non-recurrence, HCs) were included as main factors in the model. Age, sex, and interscan interval (time in days between baseline [T1] and follow-up [T2] scans) were included as covariates of no interest at follow-up (T2) time point, and set to zero at baseline (T1), to account for their known association with BD recurrences and GMV changes [30, 31]; TIV was not included as a covariate because each subject served as their own control. We applied a threshold of 0.1 to the absolute gray matter values as recommended by the CAT12 manual (https://neuro-jena.github.io/cat12-help/#stat_options). Cluster-level significance was set at *p* < 0.05 (two-tailed), with an initial cluster-forming threshold of *p* < 0.001, family-wise error (FWE) corrected for multiple comparisons. Weighted means of significant cluster values were extracted using the eigenvariate function in SPM as proxies for GMV, for further visualization and statistical analyses in Jamovi software (version 2.3.28) [36].

#### Exploratory and control analyses for longitudinal data

Different sets of exploratory and control analyses were performed to investigate factors associated with longitudinal GMV changes. First, to investigate the impact of BD episodes on GMV changes, we employed partial Pearson correlations (or Spearman’s rho for non-normal data) to assess relationships between the cluster value changes and the number and duration of depressive or manic episodes during the two-year interval (T2-T1). Second, to identify predictive indicators of different GMV trajectories between BD recurrence groups, we examined the associations between the number and duration of depressive or manic episodes prior to baseline (T1) and cluster value changes during the two-year interval (T2-T1). Third, we examined the predictive role of baseline (T1) high-sensitivity C-reactive protein (hsCRP) on cluster value changes during the two-year interval (T2-T1) [37], considering the potential relationship between low-grade inflammation and brain structural changes associated with BD (Supplementary Materials and Methods S4) [14, 15]. Fourth, to assess the influence of potential confounders, we also explored the relationships between cluster value changes and variables related to disease course and severity, including the number and duration of hospitalizations [38], as well as changes in HAM-D, YMRS, GAF scores [27], and BMI [39] between baseline (T1) and follow-up (T2) in BD patients. Fifth, using ANCOVAs, we explored the influence of comorbid psychiatric diagnoses (anxiety disorder, eating disorder, alcohol abuse, cannabis abuse), remission status (according to SCID-I), familial risk (having a first-degree relative with BD, or with MDD, SCZ, or SZA considered together) on cluster value changes. Sixth, using ANCOVAs, we also explored the influence of medication use (antipsychotic, anticonvulsant, antidepressant, lithium) [9], and, seventh, medication load (using Medication load index, Sackeim scores, and chlorpromazine equivalents [CPZ]) [40–43].

Each set of analyses was performed at a significance level of *p* < 0.05 (two-tailed), Bonferroni corrected for multiple comparisons. To account for the variation in the duration between scans, variables related to events occurring during the two-year interval (T2-T1), like the number of depressive episodes, were divided by the interscan interval (in days). Data normality and homoscedasticity were assessed using Shapiro–Wilk and Levene’s or Breusch–Pagan tests, and visually inspected using Q-Q- and residual plots for standardized and fitted residuals, respectively.

#### Whole-brain analyses for cross-sectional data

To explore cross-sectional associations between GMV and BD recurrence groups (BD recurrence, BD non-recurrence, HCs), ANCOVAs were performed using a full factorial design in SPM at both baseline (T1) and follow-up (T2) time points separately. These analyses assessed whether baseline (T1) GMV could predict future BD episodes or if such episodes influenced cross-sectional GMV changes (T2). Age, sex, and TIV were included as covariates. Cluster-level significance was set at *p* < 0.05 (two-tailed), with an initial cluster-forming threshold of *p* < 0.001, FWE corrected for multiple comparisons.

## Results

### Longitudinal association between BD recurrence groups and GMV change

#### Whole-brain analyses

A 3 × 2 repeated measures ANCOVA with recurrence groups (BD recurrence, BD non-recurrence, HCs) as between-subjects factor and time (baseline, follow-up) as within-subjects factor revealed one significant cluster in the right exterior cerebellum, indicating differential GMV changes across the three groups over the two-year follow-up interval (T2-T1) (*k* = 517 voxels, x/y/z = 50/-60/-50, *F*_2,118_ = 12.25 FWE cluster-level, *η*^2^_p_ = 0.172, *p* = 0.033; for results with TIV included as covariate in the model, see Supplementary Results S1).

#### Post-hoc *t*-tests of whole-brain analysis

**Fig. 1:**
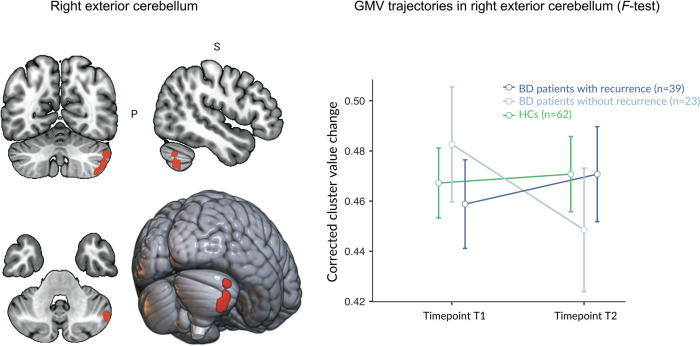
Longitudinal GMV trajectories in the right exterior cerebellum during the two-year follow-up interval between BD patients with recurrence, BD patients without recurrence, and HCs.

During the two-year (T2-T1) interval, BD patients without an episode during the two-year interval (T2-T1) showed significant GMV decreases in the right exterior cerebellum (*t*(118) = −4.24, Cohen’s *d* = 0.78, *p* < 0.001). Conversely, BD patients with a depressive or manic episode showed non-significant GMV increases (*t*(118) = 1.93, Cohen’s *d* = 0.35, *p* = 0.056), whereas HCs did not show significant GMV changes (*t*(118) = 0.72, Cohen’s *d* = 0.13, *p* = 0.471; see Fig. 1).

#### Exploratory correlation analyses in BD patients

**Fig. 2:**
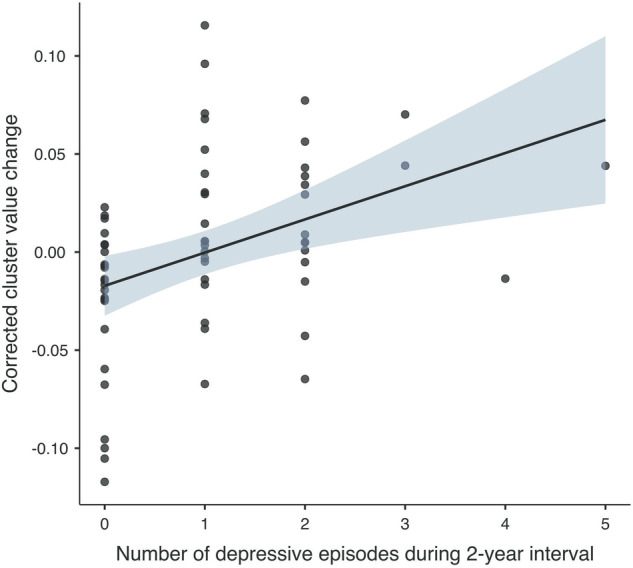
Correlation between the number of depressive episodes and longitudinal GMV changes during the two-year follow-up interval (T2-T1) in the right exterior cerebellum in BD patients.

The moderate effect size (Cohen’s *d* = 0.35) of the observed non-significant GMV increase (T2-T1) in BD patients with recurrence prompted further investigation. So, we explored the associations between GMV changes in the right exterior cerebellum (T2-T1) and the number and duration of depressive or manic episodes during the two-year interval within the overall group of BD patients. Significant associations were found between the number of depressive episodes during the two-year interval (T2-T1) and GMV increases (T2-T1) in BD patients (*rho*(62) = 0.43, *p* < 0.001; see Fig. 2). No significant correlations were found between GMV changes (T2-T1) and the number of manic episodes or the duration of depressive or manic episodes during the two-year interval (T2-T1), after adjustment for multiple comparisons (Supplementary Table S1).

#### Exploratory correlation analyses in BD recurrence groups

In BD patients without an episode during the two-year interval (T2-T1), longer durations of manic episodes before baseline (T1) time point were associated with GMV decreases in the right exterior cerebellum during the two-year interval (T2-T1) (*rho*(20) = −0.59, *p* = 0.012). This association was not significant in BD patients with recurrence (*rho*(34) = 0.31, *p* = 0.087). The duration of depressive episodes or number of depressive or manic episodes prior to baseline (T1) did not significantly correlate with GMV changes (T2-T1) in either BD recurrence group (Supplementary Table S2).

#### Exploratory linear regression analyses in BD patients

Higher baseline (T1) hsCRP levels were associated with GMV increases within the right exterior cerebellum during the two-year interval (T2-T1) in BD patients (*β* = 0.35, *t*(24) = 2.13, *p* = 0.043; Supplementary Table S3), but not in HCs (*β* = 0.30, *t*(35) = 1.60, *p* = 0.119).

#### Control analyses in BD patients

GMV changes (T2-T1) in the right exterior cerebellum in BD patients were not associated with the number and duration of hospitalizations during the two-year interval (T2-T1) as well as changes in depressive symptoms (HAM-D), manic symptoms (YMRS), global functioning (GAF), or BMI between baseline (T1) and follow-up (T2) time points (Supplementary Table S4). Similarly, comorbid psychiatric diagnoses (anxiety disorder, eating disorder, alcohol abuse, cannabis abuse), familial risk (family history of BD, or family history of MDD, SCZ, or SZA considered together), remission status (Supplementary Table S5), and medication use or medication load did not significantly influence the observed GMV changes (Supplementary Tables S6 and S7).

### Cross-sectional predictive and retrospective associations between BD recurrence groups and whole-brain GMV

No significant clusters emerged between recurrence groups and GMV at baseline (T1) and follow-up (T2) time points in the predictive and retrospective cross-sectional whole-brain analyses, respectively. This indicates that the observed GMV changes during the two-year interval (T2-T1) are likely a consequence of BD recurrences, rather than GMV alterations (T1) predicting future recurrences (T2-T1), or recurrences contributing to cross-sectional GMV alterations (T2).

## Discussion

This two-year longitudinal study illuminates the impact of depressive and manic episodes on GMV changes in BD patients within the neuroprogression framework, revealing distinct patterns of GMV changes in the right exterior cerebellum associated with mood episodes. BD patients with depressive episodes during the two-year follow-up interval exhibited GMV increases in the right exterior cerebellum, whereas BD patients without an episode experienced GMV reductions in the same area, compared to HCs. Notably, the longer BD patients without an episode had spent in a manic episode before baseline, the larger were their GMV reductions in the right cerebellum during the two-year interval. Additionally, BD patients with higher baseline hsCRP levels, compared to those with lower levels, experienced more pronounced GMV increases in the right exterior cerebellum. Importantly, all associations were independent of the number and duration of hospitalizations during the two-year interval, changes in symptom severity or global functioning between baseline and follow-up, as well as familial genetic risk, comorbid psychiatric diagnoses, remission status, and medication.

**Fig. 3:**
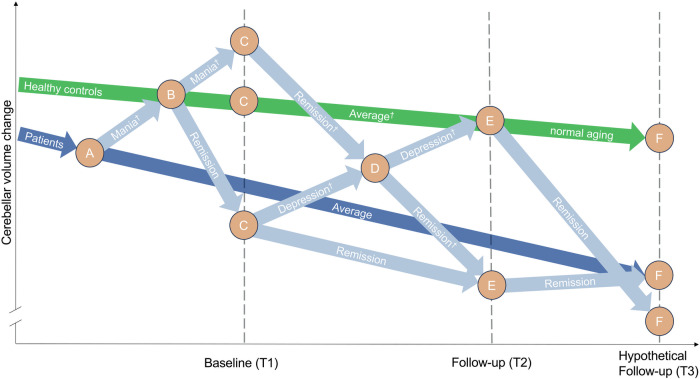
Bipolar disorder localized brain volume trajectory model. This diagram illustrates a simplified model of structural GMV changes in BD patients in relation to the timing of manic and depressive episodes, based on the findings from our study. The diagram depicts six time points (A, B, C, D, E, F) to represent potential occurrences or absences of manic or depressive episodes, with A, B, D, and F as hypothetical time points, and C and E as our actually measured MRI baseline and two-year follow-up time points, respectively. By having taken an extensive clinical history, we know the clinical states and course of illness from each patient during the times of A, B, and D. The trajectory of HCs is shown in green, individual BD patient trajectories in light blue, and the average trajectory for BD patients in dark blue. These trajectories represent expected localized brain volume change rates over time, expressed in arbitrary units to visualize approximate trends. The average patient trajectory suggests varying outcomes for different BD patient groups. Those without an episode during the T1-T2 interval might initially show increased GMV if they had experienced manic episodes before baseline, followed by a more rapid decline over the two-year interval in periods of remission compared to HCs. Conversely, those with an episode during the interval started with lower GMV at baseline but may exhibit temporary GMV increases during the T1-T2 interval. Later during remission, brain changes are thought to align with the trajectories of HCs, suggesting a normalization of previous brain changes. Over time, GMV declines more across all BD patients compared to HCs, suggesting a potential long-term impact of episodes on brain structure. This figure is adapted from and expands upon concepts of Abé et al. [9]. ^†^Indicates MRI values directly observed in our study.

Together our findings indicate a U-shaped trajectory of brain changes during the two-year interval (T2-T1) in the right exterior cerebellum in BD patients, associated with the longitudinal pattern of mood episodes. This trajectory is characterized by GMV increases during acute phases of depression and GMV decreases during periods of remission. The observed GMV decreases during remission are further intensified by past manic – but not, or to a lesser degree – past depressive episodes. The GMV increases during and/or shortly after acute depressive episodes aligned with the observed elevated baseline (T1) hsCRP levels in BD patients, suggesting an increased vulnerability of the brain to low-grade peripheral inflammation, which is linked to neuroinflammation [14, 15]. Such inflammatory processes may contribute to the observed GMV increases [44, 45], potentially through mechanisms like cellular swelling and recruitment of glial cells, particularly of microglia and astrocytes [45–49], to meet the heightened metabolic demands for maintaining brain health [50, 51]. During periods of remission, particularly in BD patients without recurrence, this glial cell proliferation may lead to maladaptive processes such as abnormal synaptic refinement or pruning [52–55], which may result in GMV reductions [56, 57]. These findings may clarify some of the contradictory findings of cross-sectional and longitudinal studies that reported GMV increases, decreases, or no changes at all in BD patients relative to HCs [9], which likely depended on the timing of the MRI data acquisition relative to the individual phase of the disorder (acute vs. remitted, manic vs. depressive, time since last episode, and duration and polarity of previous episodes). Together, these findings align with the neuroprogression model of BD, in which repeated mood episodes exert dynamic and cumulative effects on brain structure over time [11, 58]. For a visual representation of trajectory patterns across the different groups and disorder phases, see Fig. 3.

In support of this dynamic pattern, cross-sectional MRI studies demonstrated GMV increases in the cerebellum in first-episode BD patients, while GMV decreases were observed in BD patients with a history of multiple episodes and longer illness durations [59–64]. Furthermore, longitudinal studies revealed that an increasing number of depressive episodes during the study interval correlated with progressive GMV increases in the cerebellum [20], whereas an increasing number of manic episodes correlated with GMV decreases in this area [21]. While we did not observe GMV decreases with an increasing number of manic episodes during the two-year interval, we found that a longer duration of manic episodes prior to baseline was associated with more pronounced GMV decreases during the two-year interval in the cerebellum in BD patients without recurrence. This finding suggests that mania, relative to depression, may be linked to longer-lasting and/or more severe maladaptive changes in cerebellar structure that even persist into periods of remission, possibly due to the pronounced neurophysiological disruptions associated with manic episodes (see Fig. 3) [9].

These maladaptive processes may particularly occur in an area like the cerebellum [65, 66], a region increasingly recognized for its plasticity [67], critical role in emotion regulation [68, 69], and involvement in affective disorders [70]. The cerebellum is closely linked to the basal ganglia and receives input from various cortical areas, including the prefrontal and temporal cortex, to facilitate automatic behaviors [71]. The exterior part of the cerebellum is particularly involved in emotional processes [72]. Alterations in this area could disrupt functional connectivity networks of cortico-cerebellar circuits, particularly with the prefrontal cortex [73–75], which may contribute to the emotion dysregulation often observed in BD patients [64, 71, 76].

The observed GMV reductions in BD patients without recurrence contrast some previous longitudinal studies that reported no gray matter changes during periods of remission [17–19]. Beyond the timing of the MRI acquisition and the relative phase of the disorder (as illustrated in Fig. 3), these discrepancies may be due to different methodological approaches and participant characteristics. First, Abé et al. [18] did not include a control group [18], whereas our study used a matched group of HCs by age and sex to BD patients, which serves as a comparative baseline of normal age-related brain structural changes, allowing for more sensitive detection of disorder-specific brain structural changes that might otherwise be obscured in patient-only samples. Second, Abé et al. [18, 19] used Freesurfer to assess cortical thickness within larger, predefined regions-of-interest (ROIs) [18, 19], while our study utilized voxel-based morphometry to evaluate whole-brain GMV changes, allowing for the detection of subcortical and cerebellar changes that surface-based and ROI-based approaches may overlook. Third, Abé et al. [18, 19] and Kozicky et al. [17] did not account for familial genetic risk, medication load, number and duration of hospitalizations both before baseline (T1) and during the two-year interval (T2-T1), nor did they consider changes in global functioning and symptom severity (depressive or manic) during the two-year (T2-T1) interval [17–19], which are all potential risk factors for BD that may exert brain changes observed also in BD patients, that our study accounted for [27]. Fourth, our BD non-recurrence group exhibited a more severe illness course with an average of 3.6 manic episodes prior to the study, compared to 2.8 in Abé et al. [18], possibly predisposing our individuals to neuroprogressive changes even in the absence of episode recurrence during the follow-up interval. Fifth, the follow-up interval of Abé et al. [18, 19] was on average six years compared to our two-year interval [18, 19], potentially missing short-term and dynamic brain structural changes in their study. Sixth, Kozicky et al. [17] focused on first-episode BD patients [17], while our study included first- and multiple-episode BD patients, potentially exerting more pronounced neuroprogressive changes that may increase the likelihood of detecting GMV changes. Seventh, our participant demographics differed with an older average age of 40.9 years compared to 22.9 in Kozicky et al. [17], and a lower percentage of females with 52.2% compared to 61% Abé et al. [18], influencing episode recurrence and neurobiological vulnerability [30, 31]. Lastly, lower rates of medication intake with 35% on lithium in our study versus 83% in Abé et al. [18], and 48% on antipsychotics versus 75% in Kozicky et al. [17], could have exerted neuroprotective or neurotoxic effects, respectively, to stabilize or worsen GMV changes, which may have contributed to the observed brain structural differences.

Our study carefully accounted for potential confounding factors, including medication effects, familial genetic risk, substance use, and hospitalization frequency [9, 38]. Neither lithium, which is known for its neurotrophic effects and often associated with GMV increases, nor antipsychotics, which may be linked to GMV decreases, significantly influenced the observed GMV changes in our BD patients. Furthermore, familial risk, which might predispose to and modulate brain structural changes, and comorbid substance use, particularly cannabis abuse, which is associated with brain abnormalities and longitudinal brain changes, did not impact our outcomes [9]. The number of hospitalizations, as an indicator of disease severity, also showed no effect on the observed GMV changes [38]. Instead, BD episodes likely drove the observed brain changes.

Some limitations should be noted. First, while the relatively small sample size within the two BD subgroups might have limited the ability to detect modest but significant effects, large cohorts of BD patients are rare and the present study is the second largest and best characterized longitudinal MRI studies of BD patients to date [3, 9]. Second, although BD episodes were self-reported, which could introduce recall bias, we employed the life chart method to improve the reliability of the self-reported measures [77, 78]. Third, our HC group included “very healthy” participants without subclinical symptoms or familial risk for BD, which may limit the generalizability of our findings; however, the inclusion of such HCs is crucial to detect small yet significant effects, as often observed in psychiatric MRI studies. Fourth, although we accounted for several confounders, including current and past disease severity, medication, genetic familial risk, and comorbid psychiatric diagnoses, other factors such as subclinical symptoms may have influenced our findings, precluding causal inferences. Lastly, although the lack of findings in our cross-sectional, between-group predictive and retrospective analyses suggest that episode recurrence likely drove the observed GMV changes, we cannot ultimately determine the directionality of the relationship between episode recurrence and GMV changes using two imaging time points.

Our findings provide insights into the neurobiological consequences of BD episodes on gray matter over a two-year follow-up interval. The observed brain structural increases may reflect mechanisms like neuroinflammation during acute phases of BD, whereas brain structural decreases may be linked to processes like abnormal synaptic refinement or pruning during periods of remission, possibly induced by neuroinflammation from previous BD episodes. These findings underscore the dynamic nature of brain changes in BD and highlight the need for a longitudinal, comprehensive approach to better understand the neurobiological mechanisms underlying the observed brain changes in psychiatric disorders. Future studies should elucidate these relationships in larger samples and at multiple assessment points using inflammatory markers.

## Supplementary information

Supplementary Online Content

## Data Availability

The data that support the findings of this study are available from the corresponding author (FTO) upon reasonable request. MATLAB (version R2017a) code was used to generate batches for whole-brain analyses within the SPM12/CAT12 toolbox and are available from the corresponding author (FTO) upon reasonable request.
