# Supplementary material for: Differential impact of manic versus depressive episode recurrence on longitudinal gray matter volume changes in bipolar disorder
Source: Neuropsychopharmacology. 2025 Aug 15;51(3):703–11. doi: 10.1038/s41386-025-02197-x (PMC12824149; doi:10.1038/s41386-025-02197-x)
Supplement: Supplementary file 1 — Supplementary Online Content [file 41386_2025_2197_MOESM1_ESM.pdf]

Supplementary Online Content

Materials and Methods S1. Further exclusion criteria for healthy controls (HCs).

Materials and Methods S2. MRI acquisition parameters.

Materials and Methods S3. Harmonization of imaging data

Materials and Methods S4. Assessment of high-sensitivity CRP (hsCRP).

Results S1. Longitudinal association between BD recurrence groups and whole-brain GMV change with inclusion of total intracranial volume (TIV) in the model

Table S1: Relationship between episode frequency and duration during the two-year interval (T2-T1) and GMV changes (T2-T1) in BD patients

Table S2: Relationship between episode frequency and duration before baseline (T1) timepoint on GMV changes during the two-year interval (T2-T1) within BD recurrence groups

Table S3: Model coefficients of relationship between hsCRP (T1) on GMV changes (T2-T1) in the right exterior cerebellum

Table S4: Relationship between clinical variables (T2-T1) and GMV changes (T2-T1) in BD patients

Table S5: Influence of comorbid psychiatric diagnoses, remission status, and familial risk (first-degree relative) on GMV changes (T2-T1) in BD patients

Table S6: Influence of medication intake at baseline (T1) and follow-up (T2) timepoints, as well as changes in medication intake between baseline and follow-up (T2-T1) on GMV changes (T2-T1) in BD patients

Table S7: Influence of medication load at baseline (T1) and follow-up (T2) timepoints, as well as changes in medication intake between baseline and follow-up (T2-T1) on GMV changes (T2-T1) in BD patients

References

Materials and Methods S1.

Further exclusion criteria for healthy controls (HCs). To minimize potential overlaps in brain structural variance between BD patients and HCs, we further excluded HCs with a positive family history of BD and those with subsyndromal depressive or manic symptoms during the two-year follow-up interval (T2-T1). These factors are known major risk factors for BD [1–3] and are associated with GMV alterations, similar to those observed in BD patients [4–6]. The life-chart method was used to identify periods of subclinical depressive or manic states [7], and only HCs without such states during the two-year interval (T2-T1) were included. Eligibility for HCs also required a 17-item Hamilton Depression Rating Scale (HAM-D) score of less than 8 [8,9] and a Young Mania Rating Scale (YMRS) score of less than 3 [10,11]. Familial risk was assessed via a questionnaire; HCs who reported a first-degree relative treated for BD were excluded.

Materials and Methods S2.

MRI acquisition parameters. T1-weighted images were acquired using a three-dimensional magnetization-prepared rapid gradient-echo (MP-RAGE) sequence to ensure high resolution with a slice thickness of 1 mm (voxel size of 1×1×1 mm) and a field of view of 256 mm. In Marburg, imaging was performed with a Tim Trio scanner using a 12-channel head matrix Rx-coil with the following parameters: TR = 1.9 s, TE = 2.26 ms, TI = 900 ms, flip angle = 9°. In Münster, a Prisma fit was used with a 20-channel head matrix Rx-coil with the following parameters: TR = 2.13 s, TE = 2.28 ms, TI = 900 ms, flip angle = 8°.

Materials and Methods S3.

Harmonization of imaging data. Following the recommendations of the ENIGMA consortium, the ComBat tool (version 1.0.1; available at https://github.com/Jfortin1/ComBatHarmonization) was used in MATLAB (R2017a) to harmonize imaging data from two different sites (Marburg and Münster) and two body coil changes performed at the Marburg site (June 2016 and August 2018). ComBat reduces site-specific variation in the data by using empirical Bayes to adjust for differences in scanners and protocols while preserving biological or clinical variation in the data [12,13]. Our dataset contained between 17 and 72 scans per site and coil configuration, meeting the minimum number recommended for robust harmonization [12]. Quality control of all images prior to harmonization ensured that registration errors had no effect on the harmonization results [14].

Materials and Methods S4.

Assessment of high-sensitivity CRP (hsCRP). HsCRP data were available from 82 participants (66%). Blood samples were collected at baseline (T1) from non-fasting participants using tubes with additives to collect heparin plasma. Samples were frozen at -80° Celsius within 1.5 hours of collection at both the Marburg and Münster sites. The Marburg samples were directly stored at the Comprehensive Biomaterial Bank Marburg (CBBMR), while the Münster samples were transported to the CBBMR for centralized processing. All samples underwent centrifugation and analysis on an AU5800 clinical chemistry analyzer (Beckman Coulter) using a CRP Latex Highly Sensitive Calibrator (Cat#ODC0027), which determines CRP turbidimetrically. This test is highly sensitive, with a detection limit of 0.08 mg/l. The normal concentration for this assay is less than 1 mg/L. Human CRP reacts specifically with antibodies bound to latex particles, generating insoluble aggregates. The absorption of these aggregates is proportional to the CRP concentration of the sample. The routine inter-assay coefficient of variation was consistently below 5%. Four participants (5%) had values below this limit but were included in the analysis. HsCRP values across the entire sample ranged from 0.08 to 33.83 mg/L (mean = 2.50, SD = 4.98, median = 1.00). Four participants were excluded from the analysis due to hsCRP values greater than 10 mg/L, which may indicate acute inflammatory processes [15].

Statistical analysis for hsCRP. In the linear regression model, covariates such as body mass index (BMI) and current smoking status (yes/no) were included in addition to the covariates of the main analyses. Nonsteroidal anti-inflammatory drug (NSAID) use was not added as a covariate because no participant was taking NSAIDs at baseline (T1) timepoint.

Results S1.

Longitudinal association between BD recurrence groups and whole-brain GMV change with inclusion of total intracranial volume (TIV) in the model. A 3x2 repeated measures ANCOVA with recurrence groups (BD recurrence, BD non-recurrence, HCs) as between-subjects factor and time (baseline, follow-up) as within-subjects factor revealed one significant cluster in the right exterior cerebellum, controlling for age, sex, interscan interval, and TIV at baseline (T1) (k=602 voxels, x/y/z=50/-60/-50, $F_{2,117}$=13.15 FWE cluster-level, η^2_p=0.184, p=.018) as well as both at baseline (T1) and follow-up (T2) (k=592 voxels, x/y/z=50/-60/-50, $F_{2,117}$=13.38 FWE cluster-level, η^2_p=0.186, p=.019).

Table S1: Relationship between episode frequency and duration during the two-year interval (T2-T1) and GMV changes (T2-T1) in BD patients

		Right exterior cerebellum
		**BD patients**
**Number of depressive episodes during interval**	*rho*	0.43*
	p	<.001
	N	62
**Number of manic episodes during interval**	*rho*	0.29
	p	.025
	N	62
**Duration of manic episodes during interval**	*rho*	0.24
	p	.071
	N	62
**Duration of depressive episodes during interval**	*rho*	0.26
	p	.048
	N	62

Note. Using partial Spearman's rho correlations, the relationship was assessed between the intensity values of the extracted clusters and the number and duration of manic and depressive episodes at $p<.013$, after correction for multiple comparisons. Results indicate that the observed GMV increases were likely because of the number of depressive episodes during the two-year interval. *Results are significant after Bonferroni correction for multiple comparisons.

Table S2: Relationship between episode frequency and duration before baseline (T1) timepoint on GMV changes during the two-year interval (T2-T1) within BD recurrence groups

		Right exterior cerebellum	
		**BD non-recurrence**	**BD recurrence**
**Duration of depressive episodes before T1**	*rho*	0.08	0.07
	p	.776	.704
	N	19	32
**Duration of manic episodes before T1**	*rho*	-0.59*	0.31
	p	.012	.087
	N	20	34
**Number of depressive episodes before T1**	*rho*	-0.07	-0.19
	p	.767	.307
	N	23	35
**Number of manic episodes before T1**	*rho*	-0.15	0.19
	p	.531	.278
	N	22	38

Note. Using partial Spearman's rho correlations, the relationship was assessed between the intensity values of the extracted clusters and the number and duration of manic and depressive episodes at $p<.013$, after correction for multiple comparisons. Results indicate that the observed GMV decreases (T2-T1) in the BD non-recurrence group were likely due to the duration of manic episodes before T1. *Results are significant after Bonferroni correction for multiple comparisons within groups.

Table S3: Model coefficients of relationship between hsCRP (T1) on GMV changes (T2-T1) in the right exterior cerebellum

			95% Confidence Interval				
**Predictor**	**B**	**SE**	**Lower**	**Upper**	***t***	***p***	***β***
Intercept	-0.13	0.10	-0.33	0.07	-1.30	.207	
Age	0.00	0.00	-0.00	0.00	0.25	.807	0.04
Sex	0.01	0.02	-0.03	0.05	0.67	.509	0.10
Interscan interval	0.00	0.00	-0.00	0.00	0.03	.979	0.00
Recurrence group	0.07	0.02	0.03	0.11	3.68	.001	0.60
BMI	-0.00	0.00	-0.01	0.00	-1.85	.077	-0.34
Smoking status	-0.00	0.02	-0.05	0.04	-0.16	.871	-0.03
baseline hsCRP	0.01	0.00	0.00	0.02	2.13	.043	0.35

Note. $F(7, 24)=3.35$, $p=.012$.

Table S4: Relationship between clinical variables (T2-T1) and GMV changes (T2-T1) in BD patients

		Right exterior cerebellum
		**BD patients**
**Number of hospitalizations during interval**	*rho*	0.17
	p	.210
	N	62
**Duration of hospitalizations during interval**	*rho*	0.20
	p	0.142
	N	61
**GAF change**	*r*	-0.14
	p	.305
	N	62
**YMRS change**	*rho*	-0.08
	p	.532
	N	62
**HAMD change**	*rho*	0.27
	p	.041
	N	62
**BMI change**	*rho*	-0.08
	p	.536
	N	59

Note. Using partial Pearson correlations, or Spearman's rho for non-normal data, the relationship was assessed between the change in intensity values of the extracted clusters (T2-T1) and the number and duration of hospitalizations as well as changes in Global Assessment of Functioning (GAF), manic symptoms (YMRS), depressive (HAMD) symptoms, and body mass index (BMI) during the two-year interval at $p<.008$, after correction for multiple comparisons. Results indicate that the observed alterations in cluster volumes were likely not because of indicators of changes in clinical variables, but instead due to BD recurrences.

Table S5: Influence of comorbid psychiatric diagnoses, remission status, and familial risk (first-degree relative) on GMV changes (T2-T1) in BD patients

		Right exterior cerebellum
		**BD patients**
**Remission status baseline**	*F*	2.29
	p	.136
	df	55
**Remission status follow-up**	*F*	4.04
	p	.049
	df	57
**Family history of BD**	*F*	1.82
	p	.183
	df	56
**Family history of MDD/SCZ/SZA**	*F*	0.15
	p	.703
	df	57
**Anxiety disorder**	*F*	2.00
	p	.163
	df	57
**Eating disorder**	*F*	0.13
	p	.723
	df	57
**Alcohol abuse**	*F*	0.58
	p	.451
	df	57
**Cannabis abuse**	*F*	1.07
	p	.305
	df	57

Note. ANCOVA results indicate that the identified cluster was not driven by psychiatric diagnoses, familial risk, or acute illness (vs. remission) at $p<.006$, after correction for multiple comparisons. Altered GMV likely occurred due to the occurrence of BD episodes.

Table S6: Influence of medication intake at baseline (T1) and follow-up (T2) timepoints, as well as changes in medication intake between baseline and follow-up (T2-T1) on GMV changes (T2-T1) in BD patients

		Right exterior cerebellum
		**BD patients**
**Baseline (T1)**		
**Antidepressants**	*F*	0.93
	p	.340
	df	57
**Antipsychotics**	*F*	1.64
	p	.205
	df	57
**Lithium**	*F*	0.79
	p	.377
	df	57
**Anticonvulsants**	*F*	0.89
	p	.349
	df	57
**Follow-up (T2)**		
**Antidepressants**	*F*	1.80
	p	.185
	df	57
**Antipsychotics**	*F*	3.01
	p	.088
	df	57
**Lithium**	*F*	1.47
	p	.230
	df	57
**Anticonvulsants**	*F*	0.03
	p	.857
	df	57
**Change (T2-T1)**		
**Antidepressants**	*F*	1.95
	p	.151
	df	56
**Antipsychotics**	*F*	0.75
	p	.479
	df	56
**Lithium**	*F*	1.14
	p	.327
	df	56
**Anticonvulsants**	*F*	3.46
	p	.038
	df	56

Note. Using ANCOVA, results indicate that medication intake at baseline (T1), follow-up (T2), or changes in medication between T1 and T2 had no significant influence on the identified cluster at $p<.013$, after correction for multiple comparisons.

Table S7: Influence of medication load at baseline (T1) and follow-up (T2) timepoints, as well as changes in medication intake between baseline and follow-up (T2-T1) on GMV changes (T2-T1) in BD patients

		Right exterior cerebellum
		**BD patients**
**Baseline (T1)**		
**Medication load index**	*rho*	0.15
	p	.271
	N	62
**Sackeim score**	*rho*	0.02
	p	.868
	N	60
**CPZ score**	*rho*	0.15
	p	.259
	N	59
**Follow-up (T2)**		
**Medication load index**	*rho*	0.13
	p	.349
	N	61
**Sackeim score**	*rho*	-0.13
	p	.404
	N	47
**CPZ score**	*rho*	0.16
	p	.230
	N	61
**Change (T2-T1)**		
**Medication load index**	*rho*	-0.12
	p	.354
	N	62
**Sackeim score**	*rho*	-0.12
	p	.386
	N	60
**CPZ score**	*rho*	-0.02
	p	.880
	N	59

Note. CPZ, Chlorpromazine equivalent. Using Spearman's rho, results indicate that medication load at baseline (T1), follow-up (T2), or changes in medication load between T1 and T2 had no significant influence on the identified cluster at $p<.017$, after correction for multiple comparisons.

References

1. Pfennig A, Leopold K, Martini J, Boehme A, Lambert M, Stamm T, et al. Improving early recognition and intervention in people at increased risk for the development of bipolar disorder: study protocol of a prospective-longitudinal, naturalistic cohort study (Early-BipoLife). Int J Bipolar Disord. 2020;8:22.
2. Salazar de Pablo G, Cabras A, Pereira J, Castro Santos H, de Diego H, Catalan A, et al. Predicting bipolar disorder I/II in individuals at clinical high-risk: Results from a systematic review. J Affect Disord. 2023;325:778–786.
3. Leopold K, Ritter P, Correll CU, Marx C, Özgürdal S, Juckel G, et al. Risk constellations prior to the development of bipolar disorders: Rationale of a new risk assessment tool. J Affect Disord. 2012;136:1000–1010.
4. Eker C, Simsek F, Yılmazer EE, Kitis O, Cinar C, Eker OD, et al. Brain regions associated with risk and resistance for bipolar I disorder: a voxel-based MRI study of patients with bipolar disorder and their healthy siblings. Bipolar Disord. 2014;16:249–261.
5. Sarıçiçek A, Yalın N, Hıdıroğlu C, Çavuşoğlu B, Taş C, Ceylan D, et al. Neuroanatomical correlates of genetic risk for bipolar disorder: A voxel-based morphometry study in bipolar type I patients and healthy first degree relatives. J Affect Disord. 2015;186:110–118.
6. Thomas-Odenthal F, Stein F, Vogelbacher C, Alexander N, Bechdolf A, Bermpohl F, et al. Larger putamen in individuals at risk and with manifest bipolar disorder. Psychol Med. 2024:1–11.

7. Post RM, Roy-Byrne PP, Uhde TW. Graphic representation of the life course of illness in patients with affective disorder. Am J Psychiatry. 1988;145:844–848.

8. Zimmerman M, Martinez JH, Young D, Chelminski I, Dalrymple K. Severity classification on the Hamilton depression rating scale. J Affect Disord. 2013;150:384–388.

9. Hamilton M. A rating scale for depression. J Neurol Neurosurg Psychiatry. 1960;23:56–62.

10. Young RC, Biggs JT, Ziegler VE, Meyer DA. Young mania rating scale. Handbook of Psychiatric Measures. 2000:540–542.

11. Young RC, Biggs JT, Ziegler VE, Meyer DA. A rating scale for mania: reliability, validity and sensitivity. Br J Psychiatry. 1978;133:429–435.

12. Fortin J-P, Parker D, Tunç B, Watanabe T, Elliott MA, Ruparel K, et al. Harmonization of multi-site diffusion tensor imaging data. Neuroimage. 2017;161:149–170.

13. Mahon RN, Ghita M, Hugo GD, Weiss E. ComBat harmonization for radiomic features in independent phantom and lung cancer patient computed tomography datasets. Phys Med Biol. 2020;65:015010.

14. Vogelbacher C, Möbius TWD, Sommer J, Schuster V, Dannlowski U, Kircher T, et al. The Marburg-Münster Affective Disorders Cohort Study (MACS): A quality assurance protocol for MR neuroimaging data. Neuroimage. 2018;172:450–460.

15. Kushner I, Rzewnicki D, Samols D. What Does Minor Elevation of C-Reactive Protein Signify? Am J Med. 2006;119:166.e17-166.e28.
